# Supramolecular Organization of Collagen Fibrils in Healthy and Osteoarthritic Human Knee and Hip Joint Cartilage

**DOI:** 10.1371/journal.pone.0163552

**Published:** 2016-10-25

**Authors:** Riccardo Gottardi, Uwe Hansen, Roberto Raiteri, Marko Loparic, Marcel Düggelin, Daniel Mathys, Niklaus F. Friederich, Peter Bruckner, Martin Stolz

**Affiliations:** 1 Department of Biophysical and Electronic Engineering, University of Genova, Genova, Italy; 2 Institute of Experimental Musculoskeletal Medicine, University Hospital of Münster, Münster, Germany; 3 Biozentrum University of Basel, Basel, Switzerland; 4 Center for Microscopy, University of Basel, Basel, Switzerland; 5 Department of Orthopaedic Surgery & Traumatology, Kantonsspital Bruderholz/Basel, Basel, Switzerland; 6 nCATS (national Centre for Advanced Tribology at Southampton), Engineering and the Environment, University of Southampton, Southampton, United Kingdom; 7 Institute for Physiological Chemistry and Pathochemistry, University of Münster, Münster, Germany; 8 Department of Orthopaedic Surgery, University of Pittsburgh, Pittsburgh, United States of America; 9 Ri.MED Foundation, Palermo, Italy; Illinois Institute of Technology, UNITED STATES

## Abstract

Cartilage matrix is a composite of discrete, but interacting suprastructures, i.e. cartilage fibers with microfibrillar or network-like aggregates and penetrating extrafibrillar proteoglycan matrix. The biomechanical function of the proteoglycan matrix and the collagen fibers are to absorb compressive and tensional loads, respectively. Here, we are focusing on the suprastructural organization of collagen fibrils and the degradation process of their hierarchical organized fiber architecture studied at high resolution at the authentic location within cartilage. We present electron micrographs of the collagenous cores of such fibers obtained by an improved protocol for scanning electron microscopy (SEM). Articular cartilages are permeated by small prototypic fibrils with a homogeneous diameter of 18 ± 5 nm that can align in their D-periodic pattern and merge into larger fibers by lateral association. Interestingly, these fibers have tissue-specific organizations in cartilage. They are twisted ropes in superficial regions of knee joints or assemble into parallel aligned cable-like structures in deeper regions of knee joint- or throughout hip joints articular cartilage. These novel observations contribute to an improved understanding of collagen fiber biogenesis, function, and homeostasis in hyaline cartilage.

## Introduction

Articular cartilage is a connective tissue that covers the ends of long bones and provides low-friction and wear-resistant joint motion. Cartilage is only 1–3 mm thin but is routinely loaded in compression, torsion and shear and allows to absorb and distribute loads generated in joint movements. Due to its compliance, it also prevents excessive loads and protects the subchondral bone from damage. These functions are engendered by two main interpenetrating suprastructural compartments, the collagen-containing fiber meshwork and the highly hydrated extrafibrillar proteoglycan-rich matrix that comprises the large, cartilage-specific proteoglycan aggrecan [1]. Both aggrecan and the collagen networks are highly ordered structures processed in a multi-step hierarchically self-assembly process. Structural changes of proteoglycans or collagens or loss of these structures may change the water content and affect tissue functional properties, which over time can damage the tissue and ultimately turn healthy into osteoarthritic cartilage. There is general agreement that the development of osteoarthritis consists of a first, basically reversible loss of proteoglycans from the hydrated gel, followed by irreversible collagenolytic degradation of the fibrils, which then results in structural disintegration of the collagen meshwork, erosive tissue loss and ultimately to catastrophic failure of the joint.

Articular cartilage comprises three structurally distinct zones distinguished by the predominant orientations of collagen fibers: (i) a surface layer representing about 10–20% of the overall thickness in which the fibers mainly run parallel to the joint surface, (ii) an intermediate layer of about 40–60% with the fibers in random orientation, and (iii) a deep layer of about 30% with fibers highly oriented along the longitudinal bone axis to anchor articular cartilage to the subchondral bone [2, 3]. These layers result into layer specific mechanical properties of articular cartilage [4]. In addition, chondrocytes are protected against compression by a so-called territorial matrix that contains a weave of small diameter fibrils oriented parallel to the cell surfaces. At some distance from the cells, the interterritorial matrix also contains large fibrils displaying a prominent banding pattern in addition to small fibrils similar to those of the territorial matrix [5]. To obtain insight in greater detail of suprastructurally distinct cartilage fibrils we have investigated their organizations by scanning electron microscopy (SEM) at improved spatial resolution. We were especially interested in the suprastructural organization of the large fibers, i.e., how these collagen fibers are formed and how they are affected during disease. By the analysis of osteoarthritic articular cartilage, we obtained insights into new aspects of the changes in fibers organization that relate to disease which point to the underlying mechanism of osteoarthritis progression.

## Results and Discussion

### Thick cartilage fibers are supramolecular composites of thin prototypic fibrils

**Fig 1:**
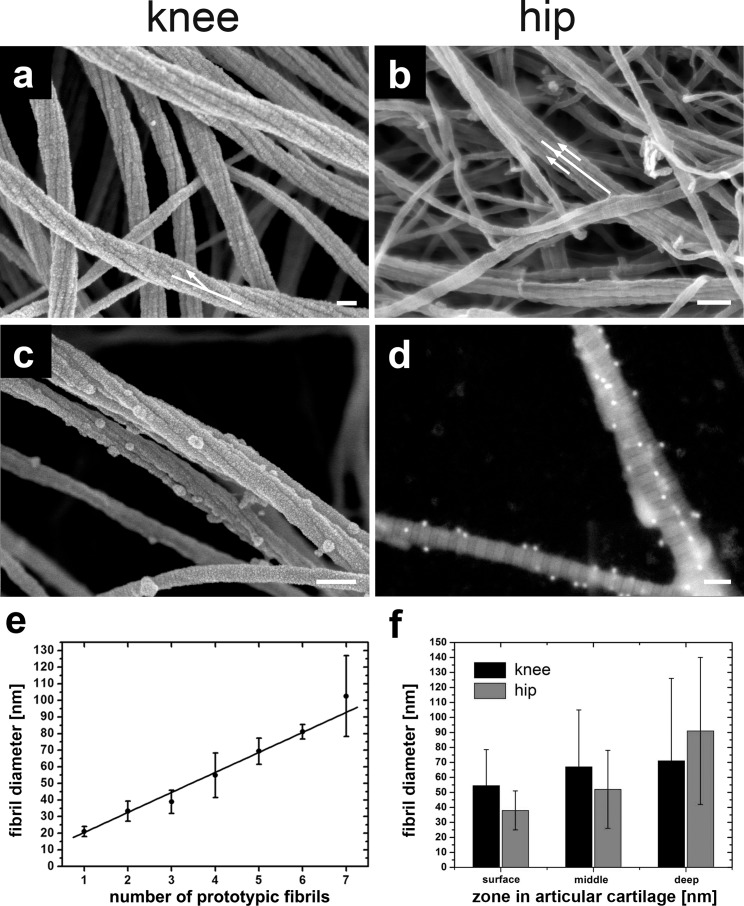
SEM images of normal human articular cartilage after enzymatic depletion of the proteoglycan moiety and chondrocytes. (a) SEM image of the collagen fiber meshwork from knee surface articular cartilage shows (i) the 67 nm D-band periodicity, (ii) the hierarchical organization of 5–7 threads of prototypic fibrils forming an individual collagen fiber; note that each prototypic fibril exhibits the 67 nm D-band periodicity; and (iii) a twisting of the prototypic fibril along the long axis of roughly about 400 nm (white arrow). (**b**) SEM image of hip surface articular cartilage with untwisted fibers (white arrows). (**c**) Collagen fibers were labeled by collagen II antibodies with 18-nm gold particles attached and directly inspected in cartilage by SEM. (**d**) imaging of 18-nm gold particles using the backscattering electron (BSE) mode in the SEM on extracted collagen type II fibers. (**e**) Graph shows the increase of collagen fiber diameter with the number of prototypic fibrils. (**f**) Comparison between fiber diameters in each zone in hip articular cartilage and knee articular cartilage. Scale bars, 100 nm (a to d).

**Fig 2:**
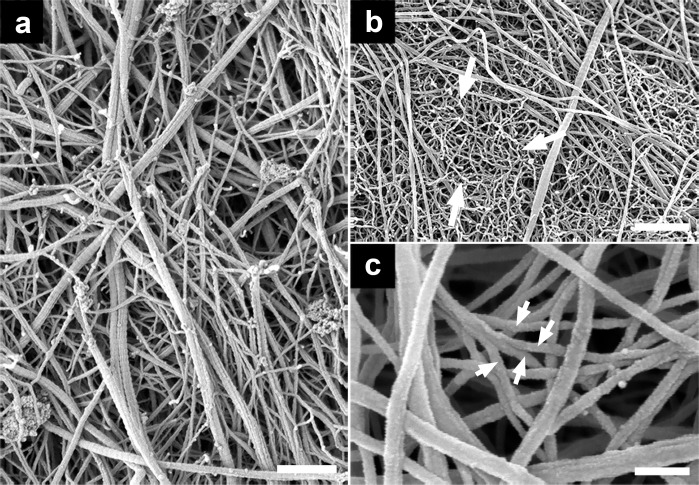
SEM images providing an overview of articular cartilage of patients undergoing knee or hip arthroplasties from osteoarthritic human articular cartilage after enzymatic depletion of the proteoglycan moiety and chondrocytes. (a) SEM image of grade 3 osteoarthritic cartilage (knee), exhibiting breakdown of thicker collagen fibers with a diameter of 40–60 nm into thinner fibers down to bundles made of only one prototypic fibril of 18 ± 5 nm in diameter. (b) SEM image of grade 3 osteoarthritic cartilage (knee) shows the end-stage of fiber breakdown, that is a wool-like structure (white arrows) with filaments exhibiting a diameter of d = 13 ± 2 nm. (c) Degrading articular cartilage larger fibers split into smaller sized fibrils that are often arranged as a highly entangled fibrillar meshwork (white arrows). Scale bars, 500 nm (a and c); 100 nm (b).

First, we inspected the surface, middle and deeper zones from normal human knee and hip articular cartilage with no signs of osteoarthritic damage (grade 0; see Materials & Methods). The surface layers from, both, knee (Fig 1A) and hip joints (Fig 1B) contained collagen fibril bundles [6] of variable diameters. The small prototypic fibrils forming the fibril bundle have a uniform diameter of 18 ± 5 nm (n = 504, R^2^ = 0.95) and present a clearly visible periodic D-banding (D = 67 ± 2 nm) (Fig 1A, 1B and 2A). Since small diameter fibrils are assembled with their D-bands in register, the larger fibril bundles appear to have a D-periodicity. We consider such small diameter fibrils as prototypic cartilage fibrils since very similar fibrils are typically found in embryonic or developing, immature avian and mammalian cartilage. Hyaline cartilage fibrils are macromolecular amalgamates of collagens II, IX, and XI [7] and can be reconstituted *in vitro* from appropriate mixtures of the same collagens in solution [8], but not by collagen II alone. Strikingly, however, we found here that prototypic fibrils associating into large fibril bundles form a right-handed helix with a twist angle φ = 13° ± 4.4° (n = 99, R^2^ = 0.75) specifically in the superficial layers of knee joint cartilage, but not in the deeper layers in knee- or, altogether, in hip joints. In the latter, bundles are formed of parallel, seemingly similar prototypic fibrils without twist (compare arrows in Figs 1A and 1B).

### Immunolabeling and SEM imaging of osteoarthritic cartilage

The quantitatively major collagen type in hyaline cartilage, including articular cartilage, is collagen II that, however, forms stoichiometrically defined amalgamates with the minor collagens IX and XI. If damaged cartilage is repaired at all, the newly formed tissue usually is fibrous cartilage containing collagen I. To directly identify the type of collagen within fibril bundles of Outerbridge grade 1 osteoarthritic cartilage, we employed immunolabeling using gold-conjugated antibodies. Specimens were first analyzed by transmission electron microscopy (TEM) to examine whether hyaluronidase and/or trypsin causes a reduction of accessible binding epitopes (see Materials & Methods). As shown in Fig 1C, the epitopes for immuno-gold conjugates for collagens I or II were resistant against hyaluronidase- and/or trypsin treatment. We then performed single and double labeling for collagens I and II in articular cartilage of grade 1 osteoarthritic knee and hip joints imaged by SEM (not shown). No labeling for collagen I was detected in double labeling experiments. As documented in Fig 1D and to assure that the dots represent gold particles we employed backscattering electron (BSE) mode showing material contrast in the SEM were we clearly identified the 18-nm gold particles. S1 Table (see Supplementary Information) provides an overview of the control experiments. Taken together, we conclude that grade 1 osteoarthritic knee (Fig 1A) and hip cartilage (Fig 1B) only contained collagen II fibrils, but not collagen I.

### SEM measurements of increasing fibril bundle diameters with number of prototypic fibrils agree with diffraction data

As shown in Fig 1E, the diameters of fibril bundles from, both, knee and hip joints are plotted as a function of the number of their smaller prototypic fibrils subunits. Smaller fibril bundles exhibit between 1 and 3 prototypic fibrils as supramolecular subunits. In large fibril bundles of deep zones the number of prototypic fibrils can be up to 10. These observations are consistent with a progressive, step-wise increase in diameters as previously demonstrated in tendons and ligaments by TEM [9]. This is also supported by the work of Antipova and Orgel [10] which could show that thick fibers are in fact bundles of thin fibrils. They postulate that a “thin-fibril” is an irreducible collagen fibril (without enzymatic digestion or mechanical force) formed from closely packed collagen molecules and held together through collagen-collagen interactions such as lysine-hydroxylysine bonds. Our observations are inconsistent, however, with the notion of lateral growth of cartilage fibrils by apposition of individual collagen molecules to form prototypic fibrils. Our data suggest that the mode of lateral packing must be interpreted as a consequence of lateral fusion of prototypic fibrils during the process of maturation. This interpretation is consistent with a continuous, progressive, step-wise increase of cartilage fibril diameter as previously been described for tendons and ligaments by TEM and interpreted as a consequence of lateral fusion of prototypic fibrils during fibril maturation [9]. The average slope of the fitted data in Fig 1E intersects at 20 ± 2 nm for the first prototypic fibril. The diameter of fibril bundles then increases with the addition of prototypic fibrils exhibiting a slope of 12 ± 1 nm in excellent agreement with the diffraction data by Orgel et al. [11] and with the findings based on scanning transmission electron microscopic mass mapping by Holmes and Kadler [12]. Fig 1F shows an increase of fiber diameters with depth in the knee and hip joints. In both joints the spread of fiber diameters increases with depth exhibiting larger diameters but also an increasing amount of fibrils with a smaller diameter between 20 nm and 40 nm. Hip articular cartilage also exhibits a larger range of collagen fiber diameters compared to knee articular cartilage.

### Damage to collagen fibers in osteoarthritic cartilage

**Fig 3:**
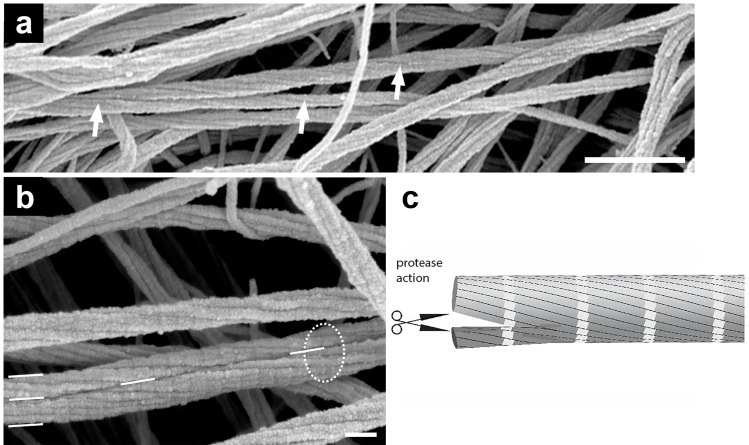
SEM images of grade 0 knee and hip articular cartilage show the breakdown of cartilage at the individual fiber level. (a) SEM image of grade 0 articular cartilage (knee) shows a fiber that splits apart and forms two new perfect but smaller diameter fibers as indicated by the arrows. (b) SEM image of grade 0 articular cartilage hip cartilage exhibiting prototypic fibrils that are arranged in register. The thick fiber at the bottom of the image (white circle) has split into two fibers of smaller diameters. (c) Scheme suggesting that the splitting of fibers is induced by proteases. Scale bars, 500 nm (a); 100 nm (b).

The extent of structural damage of articular cartilage was determined using the Outerbridge grading scale (see Materials & Methods). Advanced stage osteoarthritis is associated with a progressively increased disruption and loss of collagen fibers that is counterbalanced with remodeling of the collagen meshwork. The disruption of the fibers leads to a loss of the proteoglycans [13, 14] which then accelerates the damage of cartilage. We first provide electron micrographs at low magnification of disrupted collagen fibers. Fig 2A shows a distribution of different diameters of collagen fibers as documented here with grade 3 osteoarthritic cartilage. As shown in Fig 2B, some of the thin filaments with no obvious D-banding and with a mean diameter of 13 ± 2 nm, (n = 255, R^2^ = 0.89). These thin filaments exhibit a higher curvature (lower persistence length) and were highly entangled in comparison with the larger diameter collagen fibers in, for instance, Fig 1A and 1B. Fig 2C shows examples of unwinding composite fibers into thin filaments in osteoarthritic cartilage. Next, we want to show collagen breakdown at the single fiber level. There is a strong agreement that one of the first signs of aging in cartilage is related to a continuous loss of its proteoglycan moiety, i.e. osteoarthritis first affecting the PGs, followed by changes in the supporting collagen meshwork [3, 15–17]. Interestingly, Fig 3A exhibits splitting of collagen fibers in articular cartilage in grade 0 OA (white arrows). Therefore, this disassembly of collagen fibers occurs at a relatively early stage of disease when the articular cartilage surface was apparently smooth and intact. Splitting of composite collagen fibers into prototypic subunits increased for higher grades of osteoarthritis. Fig 3B also depicts a collagen fiber that has just been split into two separated smaller sized collagen fibrils (visualized by the white dashes). Note that the prototypic fibrils are still aligned in register with respect to the original collagen fiber (enclosed by the dashed circle), but the prototypic fibrils have already completely separated into two new separate fibrils. Therefore, we propose a two-step process for the splitting of larger collagen fibers: in a first step, individual collagen molecules of a thicker fiber are cleaved by proteolytic enzymes which results in two smaller composite fibers (fibril bundle). In a second step, the two daughter collagen fibrils (fibril bundle) unwind and/or separate into new prototypic fibrils. Such a disruption becomes possible since mature fibers of interterritorial cartilage matrix are formed by fusion of prototypic ~ 18 nm wide fibrils also present throughout cartilage matrix but particularly abundant in territorial zones of mature joint cartilage or in embryonic cartilage [8]. Low resolution EM data have led to the speculation that prototypic fibrils can fuse into larger fibrils, particularly in the interterritorial regions of the deep zones in articular cartilage [5].

**Fig 4:**
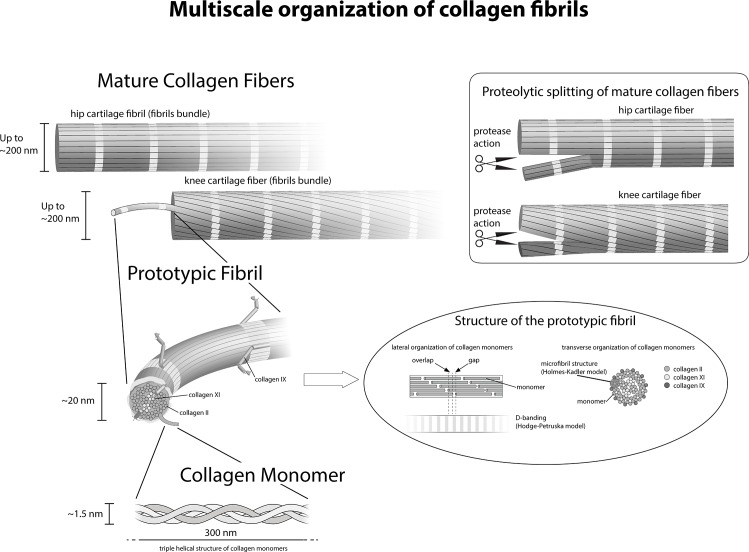
Schematic representation of fiber formation and fiber breakdown. Three identical [α1(II)]_3_ chains assemble into a right-handed triple helix and form a collagen type II monomer that is approximately 300 nm long and 1.5 nm in diameter. In the next step of hierarchical organization collagen monomers assemble into highly ordered 67-nm staggered arrays of collagen monomers. The Hodge & Petruska model (Petruska and Hodge, 1964) explains this characteristic D-periodic banding formation. A collagen fiber in the knee surface zone is composed of threads of smaller fibrils that we identified as prototypic fibrils. Each individual collagen-containing fiber is assembled of multiple single prototypic fibrils. The prototypic fibrils are aligned in register. Schematics (upper right corner) explaining the splitting of fibers and formation of smaller fibers: Collagen fibers in the knee or hip surface zone after splitting into two perfect collagen fibers of smaller diameters. The prototypic fibrils are aligned in register, in the original fiber as well as in the daughter fibers.

We have documented that collagen fibers are organized like multi-thread ropes in which each thread corresponds to a prototypic sub-fibril. Such multi-thread ropes are more flexible than compact fibers of the same diameter and therefore better suited for soft deformable materials or structures such as articular cartilage. Furthermore larger fibrils present an increased safety against failure known in rope fiber industry as the Cook-Gordon effect, and describes that an occasional crack in a multi-thread cable is diverted and stopped at the surface of each thread and does not propagate through the whole diameter as it would in a single-thread rope [18]. It is evident that the rope-like 3D structure in Figs 1A, 3A and 3B, shows that the alignment in register requires an increase of length for the outer prototypic fibrils and the collagen molecules to stretch without damage. We have not observed fibers that were composed of more than 10 prototypic fibrils. This finding suggests that the increasing tension of the outer prototypic fibrils may be a—or even the—limiting factor for fibril growth in the twisted fibers. The Hodge & Petruska [19] model explains the banding pattern of 67 nm in 2D (Fig 4). However, the fibers are 3D structures that with an increasing diameter of the “rope” obviously need to be stretched to fall in register. This stretching of the prototypic fibrils must be also important for all deformations of the collagen fibrils nanostructure and should be included when modeling fibers.

The knee is a hinge joint, whereas the hip is a ball and socket joint. Both joints represent different mechanical loading conditions during joint motion and, therefore, different strains and shear stresses are exerted to the articular cartilage surfaces. In normal gait, both joints are cyclically compressed at a frequency of ~ 1 Hz. The knee joint is not pre-stressed and therefore the articular cartilage experiences local variations of forces that range from unloaded cartilage up to local forces exerted that are multiple times the bodyweight. By contrast, the femoral head due to its encapsulation into the acetabulum is under constant pre-stress load. In addition, the local forces applied during gait are distributed over a larger area hip articular cartilage than in the distal femur. The most important function of the collagen fibers in both joints is to contribute to the high tensile forces and to protect the articular cartilage from rupture. Whereas helical ropes can respond more favorably to higher stresses before breaking, a parallel alignment of individual threads within a cable is better suited for constant pre-load condition [20, 21].

Insights into the details of the assembly process of composite fibril bundles starting from prototypic fibrils will lead to an improved understanding of normal cartilage function, development, remodeling and degeneration. For example, it will be interesting to learn how the steric hindrance of collagen IX is eliminated before fusion can occur. In mature cartilage, the ECM can be differentiated into territorial regions near chondrocytes and more remote, interterritorial regions. Territorial regions almost exclusively contain thin, prototypic fibrils composed of collagen II, IX, XI. By contrast, thick and strongly banded fibers without collagen IX, but coated with decorin, occur in interterritorial regions together with small quantities of prototypic fibrils [22]. It is tempting to speculate, therefore, that collagen IX is proteolytically degraded in prototypic fibrils under way to fusion into large cartilage fibrils bundles. The mechanism of alignment of prototypic fibrils in D-periodic register preceding their fusion is also still unknown and will be subject to further investigations. However, preliminary experiments suggest that decorin orchestrates the formation of large, tension-resisting cartilage fibers whereas collagen IX stabilizes small prototypic fibrils and their interfibrillar separation. Collagen IX may stabilize three-dimensional fibril networks by incorporation of discrete collagenous domains into more than one fibril (unpublished results). This information will also be the foundation for the establishment of the appropriate fiber assembly in cartilage tissue engineering.

A possible mechanism for a non-enzymatic decomposition of collagen fibers at least for rheumatoid arthritis is proposed by Antipova and Orgel [10]. Their work suggests that the effect of antibodies against biglycan is more potent than harsh chemical and/or enzymatic treatments designed to mimic arthritis-like fibril de-polymerization. In the presence of this biglycan antibody, thick collagen II containing fibers are quickly decomposed into much smaller, probably prototypic collagen fibrils. However, in the case of osteoarthritis, plausible processes for the decomposition of thick fibers into smaller prototypic fibrils are still unknown. Possible mechanisms include the “peeling” of outer layers of the fibers which would open up binding sites for cell adhesion receptors, as well as collagenase cleavage sites [23]. In addition, the results of Perumal and co-workers also suggest that the C-terminal telopeptides must be proteolyzed before collagenase can gain access to the cleavage site.

In conclusion, we have shown using high resolution SEM that the large fibers in articular cartilage are constituted by bundles of small, homogeneous prototypic fibrils aligned in register, with tissue-specific organizations at different anatomical sites. Furthermore, our data suggests that fiber breakdown during osteoarthritis initially occurs by “unbundling” of prototypic fibrils suggesting a potential target for therapy. These novel observations contribute to an improved understanding of collagen fiber biogenesis, function, and homeostasis in hyaline cartilage and on fiber hierarchical organization in health and disease (Fig 4). The schematics of Fig 4 combines our finding with the axial organization of collagen monomers from the classic Hodge-Petruska model [19] and their lateral organization schematized by Kadler et al. [24]. This schematics simplifies a number of aspects for the sake or representation, hence presenting some limitations, in particular in the structure of microfibrils within collagen prototypic fibrils that is more precisely described by Orgel et al. [6]. When coupled with other high resolution analytical techniques [25–28] and high throughput screening tools [29, 30], our findings could help identify the events involved in disease progression as well as in development, thus providing new, crucial clues for both disease modifying osteoarthritis drugs and more effective tissue engineering approaches.

## Material & Methods

### Human articular cartilage specimens

Osteoarthritic articular cartilage was obtained from patients undergoing total hip (five) or knee (eleven) arthroplasty. In addition, we used control cartilage from two cadavers (knee) and from two patients with femur neck fracture exhibiting no signs of osteoarthritis. The cartilage used in these studies were taken from the cut out parts of the joints of patients undergoing arthroplasties and considered as waste material. The ethics committee of the respective institutions waived need for further consent. The specimens were stored in ice-cold phosphate-buffered-saline (PBS, pH 7.0) supplemented with a protease inhibitor cocktail (Complete; Boehringer Mannheim,) and 0.01% NaN_3_. Each individual specimen was graded independently by two experienced clinicians using the Outerbridge classification scale [31]: grade 0 represents undamaged, “healthy” cartilage, grade 1 describes articular cartilage with softening and superficial lesions, grade 2 refers to partial-thickness defects with fissures down to < 50% of the cartilage depth, grade 3 describes severe damage with fissuring reaching the subchondral bone, while grade 4 refers to fully exposed subchondral bone with no significant articular cartilage left. From each area presenting a clear Outerbridge grade from 0 to 3, three adjacent osteochondral plugs (2 mm in diameter) were obtained using a clinical punch biopsy tool. Specimens were stored on ice in PBS and prepared on the same day for SEM imaging. Specimens for immunoblotting were extracted and frozen at -20°C until use. As control for immunolabeling with collagen I antibodies, we obtained ligaments from two human donors, as well as from porcine and bovine harvested within 2 hours after slaughter. For SEM imaging of the fibrils in the deeper layers of the tissue, three articular cartilage samples from knee and three from hip from different donors, all graded as 0 on the Outerbridge scale, i.e. with no obvious sign of osteoarthritis, were cut with a scalpel along the cartilage loading axis prior to glutaraldehyde fixation (see below).

### Scanning Electron Microscopy

Following the method of Segawa and Takiguchi [32], proteoglycans were extracted from articular cartilage specimens in 100 mM Soerensen’s phosphate buffer (pH 7.2) containing 1mg/ml bovine hyaluronidase (type I-S Sigma, St Louis, MO, USA) and 1mg/ml trypsin (type I, Sigma, St Louis, MO, USA) and 0.5% sodium azide for 3 days at 37°C. The solution was renewed every 24 h. Specimens were then fixed with a 2.5% gluteraldehyde (in PBS, 2.6 mM NaH_2_PO_4_, 3 mM Na_2_HPO_4_, 155 mM NaCl, 0.01% NaN_3_ w/v, pH 7.2) for 2.5 h at room temperature. Next, they were vortexed three times in ultrapure water and dehydrated in an ascending ethanol series up to 100%). After critical point drying, samples were sputter-coated with 3–5 nm of platinum and examined by SEM (Hitachi S-4800 FEG) operated at 1.5–5 kV accelerating voltage. Areas corresponding to joint surface were selected for imaging, choosing flat, uniform parts, and avoiding any macroscopic defects as well as the territorial and pericellular envelope of surface chondrocytes.

### Immunolabeling and TEM

10-μl aliquots of extracts containing suprastructural fragments were spotted onto sheets of parafilm. Nickel grids coated with formvar/carbon were floated on the drops for 5 min to allow adsorption of aggregates and were washed with distilled water. For transmission electron microscopy, collagen fibrils on grids were negatively stained with 2% uranyl acetate for 10 min. For immuno-gold electron microscopy, grids were treated for 30 min with 2% (w/v) dried skim milk in PBS and, subsequently, for 2 h with the same buffer containing antiserum to collagen XI or decorin, polyclonal antibodies to collagen I or monoclonal antibodies to collagen II (dilution 1:100 and 1:400, respectively). After extensive washing with PBS, the grids were put on drops of 0.2% dried skim milk in PBS, containing colloidal gold particles (18 nm) coated with goat antibodies to rabbit immunoglobulins at dilutions recommended by the manufacturer (Jackson Immuno Research). Finally, the grids were washed with distilled water and negatively stained with 2% uranyl acetate for 10 min. Negative controls were done without first antibody treatment. Electron micrographs were taken at 60 kV with an EM 410 electron microscope (Philips).

### Immunolabeling and SEM imaging

For each donor presenting all grades of osteoarthritis, prior to glutaraldehyde fixation, two adjacent samples from areas graded from 0 to 3 on the Outerbridge scale have been rinsed in PBS and immersed in skimmed milk solution (2% in PBS) for 30 minutes and incubated overnight at 4°C in a solution with both primary antibodies diluted 1:50 in a 0.2% solution of skimmed milk in PBS. Next, samples were gently rinsed three times with PBS and incubated with both secondary antibodies (dilution 1:10 in a 0.2% w/v solution of skimmed milk in PBS) for 3 hours at room temperature. For each couple of adjacent samples, the first one was incubated with a solution of both the secondary antibody conjugated with 30 nm gold particles to identify collagen I, and the secondary antibody conjugated with 18 nm gold particles to identify collagen II. Negative controls were done with the first antibody omitted. Next, samples were rinsed in PBS and prepared for SEM as described in the above paragraph.

For immunolabeling, rabbit polyclonal antibodies to collagen I (R1038, Acris Antibodies, Hiddenhausen, Germany) and mouse monoclonal antibodies to collagen II (MAB8887, Millipore), were used as primary antibodies. As secondary antibodies peroxidase-conjugated polyclonal goat anti-mouse immunoglobulins (Dako Cytomation) and peroxidase-conjugated polyclonal anti-rabbit immunoglobulins (Sigma-Aldrich) were used. As labels we used 18- and 30-nm colloidal gold particles (Jackson ImmunoResearch) alternatively conjugated to anti-mouse or anti-rabbit immunoglobulins. Antibody cross-reactivity was assessed by immunoblotting, resulting in no cross-reactivity for MAB8887 and a weak signal against collagen II for very high concentrations of the antibodies against collagen I (R1038).

### Statistical analysis

Thicknesses of collagen fibrils and twisting angles were measured using the software provided with the SEM (ICW (2.0); Boerder Elektronik, Germany). All values of collagen fibril diameters are given as mean ± standard deviation (SD) of their distributions. Significance was tested using the Kruskal-Wallis test (p < 0.05).

## Supporting Information

S1 Fig TEM image of immunogold labelled collagen fibril extracted from healthy human articular cartilage after treatment with hyaluronidase and trypsin. Representative example of a collagen II labelling. Bar = 100 nm.
(TIF)

S1 Table Analysis of enzyme treatment on human AC and bovine tendon. (TIF)
