# Supplementary figures and images for: Supramolecular Organization of Collagen Fibrils in Healthy and Osteoarthritic Human Knee and Hip Joint Cartilage
Source: PLoS One. 2016 Oct 25;11(10):e0163552. doi: 10.1371/journal.pone.0163552 (PMC5079628; doi:10.1371/journal.pone.0163552)

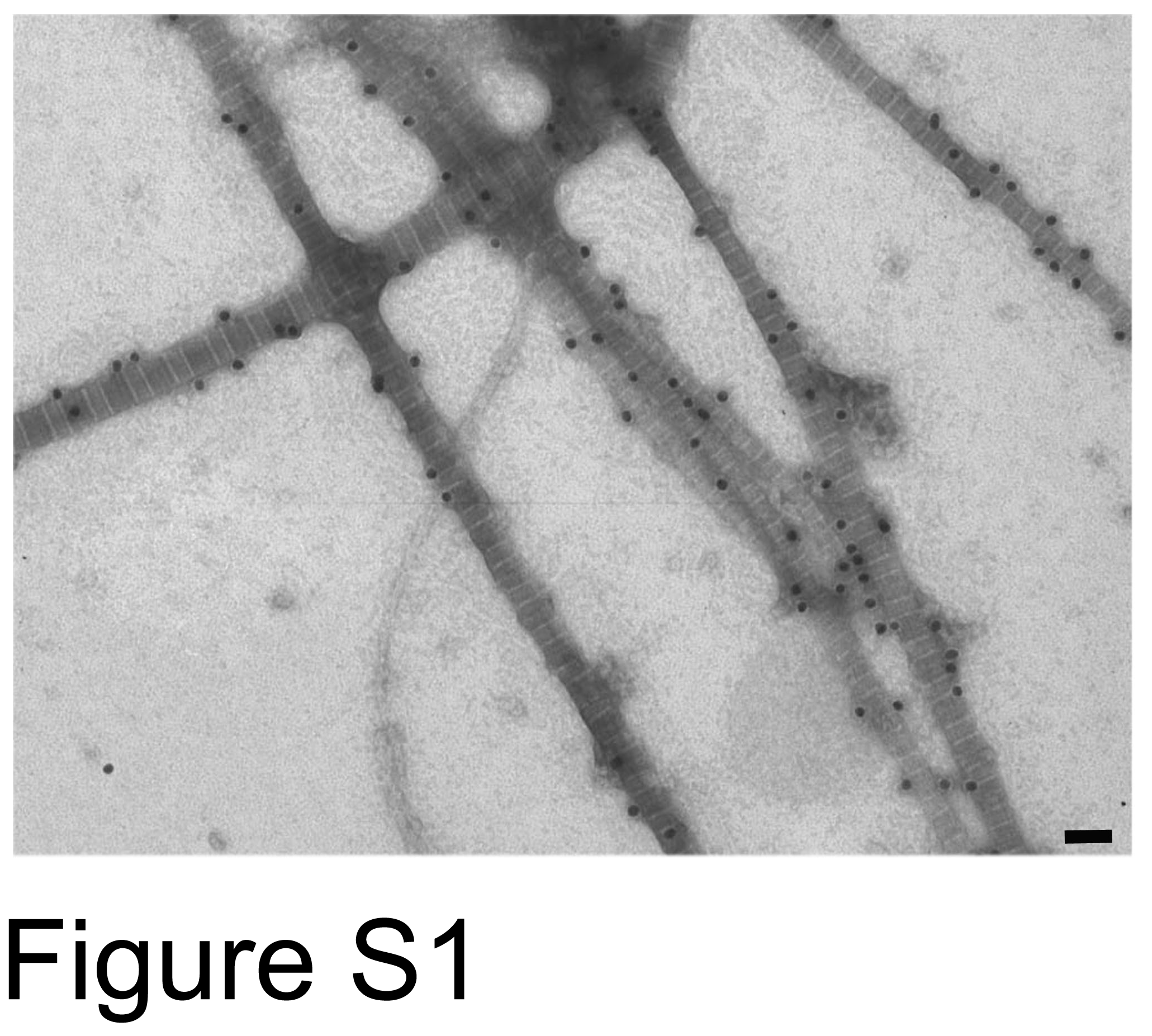

Supplement: S1 Fig — Representative example of a collagen II labelling. Bar = 100 nm. (TIF) [file pone.0163552.s001.tif]

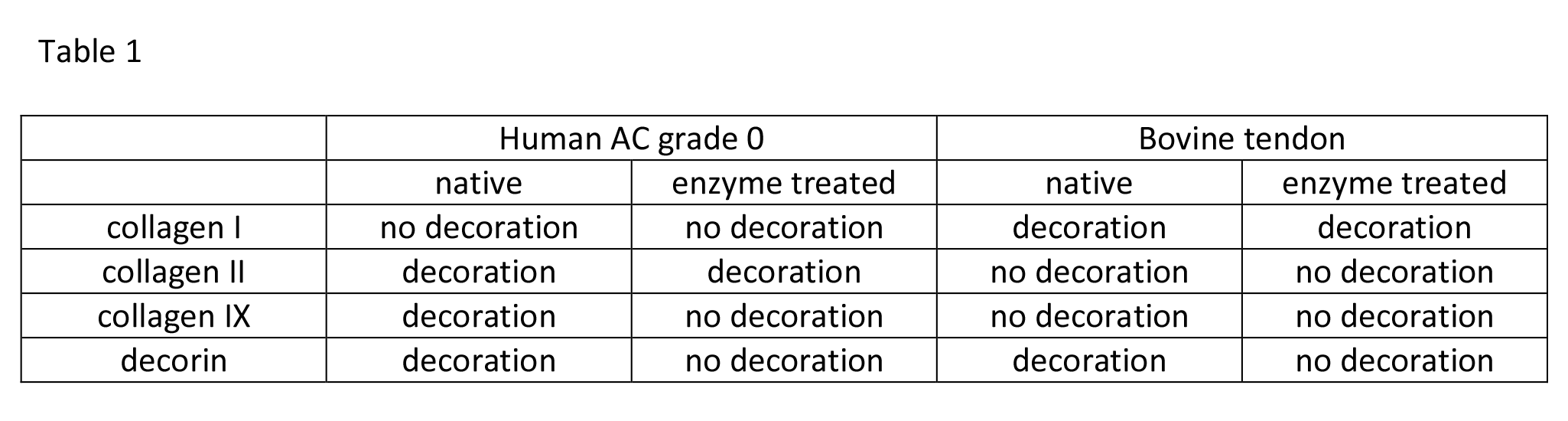

Supplement: S1 Table — (TIF) [file pone.0163552.s002.tif]
